# The Impact of Utilizing a Balancing Blindfold During Training on the Backward Running Technique in Experienced and Novice Male Handball Players

**DOI:** 10.3390/biomimetics10100649

**Published:** 2025-09-28

**Authors:** Aydin Najipour, Siamak Khorramymehr, Kamran Hassani

**Affiliations:** 1 Department of Biomedical Engineering, SR.C., Islamic Azad University, Tehran 1477893855, Iran; 2 School of Mechanical, Industrial and Aeronautical Engineering, Witwatersrand University, Johannesburg 2050, South Africa

**Keywords:** bionics, blindfolded balancing, handball, handball action, kinematics, backward running

## Abstract

Backward running is common in handball defense and relies heavily on proprioceptive control when visual information is limited. Twenty-eight male handball players were allocated to three groups: experimental novice group with blindfold training (*n =* 7), control novice group with the same training without blindfold (*n =* 7), and target professional group (*n =* 14). Both novice groups completed a 6-week balance program (3 × 20 min/week). Lower-limb kinematics during backward running were captured with a 6-camera motion analysis system, and inter-joint coordination was quantified by Mean Absolute Relative Phase (MARP) and Deviation Phase (DP) for ankle–knee and knee–pelvic couplings. At baseline, professionals showed greater ankle–knee MARP than novices (ANOVA F(2,25) = 9.42, *p* < 0.001). Representative means (mean ± SD): ankle–knee MARP novices 1.62–1.79 vs. professionals 3.83. After training, ankle–knee MARP increased in both novice groups (experimental: t(6) = 4.72, *p* < 0.001; control: t(6) = 5.02, *p* < 0.001), approaching professional values (post-training novices ≈ 3.22–3.26). Post-training between-group differences were non-significant for ankle–knee MARP (ANOVA F(2,25) = 1.24, *p* = 0.30), while ankle–knee DP showed a group effect (F(2,25) = 5.12, *p* = 0.01; experimental vs. professional t(19) = 3.12, *p* = 0.01). A short-term balance program improved ankle–knee coordination during backward running in novice male players; additional blindfolding did not yield extra benefit over 6 weeks. These findings can inform short-term training and rehabilitation planning for handball, while long-term effects require future study.

## 1. Introduction

Handball is a team sport that has been played professionally since 1946. Handball is an energetic and physically challenging sport that needs exceptional agility, strength, and balance. The incidence of injuries in this activity, similar to many other physical sports, is significantly elevated and typically entails substantial financial burdens for organizations and athletes. Recent research indicates that injury rates among youth handball players vary from 9.9 to 41 injuries per 1000 h of competition and from 0.9 to 2.6 injuries per 1000 h of practice [1].

The essential skills for this sport encompass rapid movements, backward running, and coordination under dynamic conditions. Backward running, in particular, is fundamental for defensive maneuvers, allowing players to retreat quickly while maintaining visual contact with the ball and opponents [2]. In recent years, researchers have been endeavoring to discover efficacious methods to avert the incidence of musculoskeletal injuries, taking into account the expenses and repercussions associated with such accidents in sports [3]. An approach explored in recent studies is the examination of athletes’ gait. Motion analysis can be conducted by employing instruments that quantify movement, mechanics, muscle activity, as well as the ocular and cerebral functions within the human body. Gait analysis is employed to evaluate the locomotion patterns of individuals [4].

Previous studies have applied gait and motion analysis to investigate athletes’ movement patterns and to identify factors related to injury risk. These methods provide detailed kinematic data that highlight deficiencies in inter-joint coordination and neuromuscular control. In the context of handball, analyzing gait during backward running is especially relevant, as it can reveal risk factors for knee and ankle injuries and guide the development of preventive training approaches such as blindfolded balance training [5,6].

### 1.1. Previous Studies on Backward Running in Handball

Among the various movements of handball athletes, backward movements in which the person is forward but runs backwards, and, in this case, does not have enough control over the body and the environment, the probability of injury is very high [7]. For this reason, this topic has been of great importance among researchers in this field in recent years. Chaudhary proved in his field study that backward running in handball increases the probability of injury in athletes [8]. Also, Kaparos et al. [9] proved the possibility of increased risk and injury among basketball athletes who run backward and look forward. This situation occurs when the athlete runs back in a defensive position (when the opposing team is in a counter-attacking position). In this situation, because the athlete wants to have the best reaction in front of the opposing team and track the ball’s route, he avoids looking back and goes backwards due to the depth of the playing field and the placement of other players in different locations. Scientifically, backward running has different biomechanical characteristics than running forward [10,11,12]. Proprioception, the sense of movement and joint position, plays a critical role in maintaining coordination during dynamic skills such as backward running. Under conditions of visual deprivation, such as blindfolded training, athletes rely more heavily on proprioceptive cues, making this aspect directly relevant to the present study [13,14].

Effective sensorimotor integration relies on the dynamic interplay of visual, vestibular, and proprioceptive systems. While visual input often guides postural control and movement, manipulating or reducing vision can actively encourage the sensorimotor system to re-weight inputs in favor of proprioceptive and vestibular feedback, potentially enhancing motor control strategies. Recent literature highlights the pivotal role of proprioceptive enhancement within sports science and rehabilitation contexts. An editorial by Rojas-Valverde et al. [15] emphasizes the central function of proprioception for postural regulation, athletic performance, and injury prevention. In addition, a systematic review and meta-analysis by Majelan et al. [16] found that proprioceptive training significantly improves postural balance in athletes recovering from anterior cruciate ligament reconstruction, indicating that proprioceptive-based interventions yield substantial practical benefits. Moreover, research exploring sensory re-weighting mechanisms under visual deprivation has gained momentum. For example, Sung et al. [17] investigated how visual constraints influence postural sway within defined spatial boundaries, offering insight into how sensory input modulations impact stability and motor adaptation. These findings collectively support the theoretical rationale for employing balancing blindfold training in handball. By selectively depriving visual input, athletes are compelled to refine proprioceptive and vestibular coordination, which may enhance dynamic control, adaptability, and resilience during backward running—particularly in scenarios where direct visual feedback is limited.

Recently, there have been limited studies on backward running in handball players, which are described below. In a study, Sammoud et al. [18] randomly assigned 29 female handball players to three groups: forward running, backward running, and a control group. After 8 weeks of training, both training groups showed significant improvements in muscle strength, running speed, ability to change direction, and repeated running ability. However, backward running training had slightly greater improvements in 10 m running speed and change direction speed than forward running training. Uthoff et al. [19] showed in a study that backward running training can help improve forward running and vertical jump performance in adolescent athletes. Their training also helped improve running economy and cardiorespiratory function. These studies suggest that backward running training can help improve the physical performance of handball players and may have more benefits than forward running training in some aspects. Abdulrazzaq et al. [20] evaluated the effect of a specific training program on improving motor reaction speed, increasing motor satisfaction, and improving short-term defensive skills in handball players. An experimental method was used to evaluate the effectiveness of the training program in this study. This study showed that a specific training program can effectively improve the motor performance of handball players. Implementing such programs can be effective in developing defensive skills and increasing the motor satisfaction of players. Hadjisavvas et al. [21] investigated the effect of posture and body composition on stability and motor performance of professional handball players in a study titled The Importance of Posture and Body Composition for Stability and Selected Motor Abilities of Professional Handball Players. The aim of this study was to analyze the body composition, posture, and postural stability of professional male handball players and determine the differences between players with correct and incorrect posture. Also, the effect of these factors on lower limb strength, agility, speed, and directional change deficits was investigated. The researchers concluded that incorporating postural retraining exercises into training programs is essential to improve balance and motor performance of handball players. Among the joints of the body, the ankle is of particular importance due to the weight bearing of the body and the variety of movements. This joint provides movement and strength of the ankle joint with the help of soft tissue and ligaments around it. As a result, the current study examines the kinematics of the lower limbs of professional and novice athletes during movement, as backward running, with the help of the degree of coordination of movement between joints based on the phase difference, to determine the effectiveness of balance exercises using blindfolds. In other words, by determining movement indicators such as speed, linear and angular acceleration in looking forward and backward running, the correct model of backward running movement is tried to be presented to handball coaches and athletes.

### 1.2. Objectives and Hypotheses of the Present Study

Based on the theoretical background and previous findings, the following hypotheses were formulated: (1) Blindfold training will significantly enhance motor coordination in novice handball players by increasing reliance on proprioceptive feedback, as reflected in MARP indices. (2) Professional handball players will exhibit smaller improvements, since their coordination and proprioceptive abilities are already well developed. (3) Blindfold training will not provide additional benefits compared to traditional training when comparing the two novice groups (test vs. control).

The aim of this study was to form 3 different groups of novice and professional handball players in the form of experimental, control, and target group to determine the effect of using a balance blindfold and training sessions on the backward movement of handball players. Therefore, to carry out this research, the following assumptions can be considered:

a. There is a difference between the movement patterns of professional and novice backward running.
b. Fatigue affects the movement pattern of professional and novice athletes in backward running
c. The ability to control and coordinate ankle movements is different in novice athletes.
d. The motor neuron system of novice athletes works differently in dealing with the environment.

In this study, we aim to answer the following questions:

A. Can using a balance blindfold be effective in backward movement?
B. Can using training sessions be effective in improving the backward movement of novice handball players?

## 2. Experimental Procedures and Techniques

This section provides a detailed explanation of the research methodology, including topics such as the statistical sample, statistical sampling, data collection methods, measuring tools, variable measurement techniques, and statistical analysis methods.

### 2.1. Database

The educational data collection was conducted in the laboratory of physiological signal processing, located in the Faculty of Electrical Engineering at the Tehran branch of Islamic Azad University. Prior to the experiment, all participants were required to verify and endorse the informed consent form in order to partake in the experiment. The data registration for this project has received approval from the university’s ethical committee under the code IR.1401.6. According to the Cochran equation, a total of twenty-eight male athletes were involved in this experiment, divided into three distinct groups. The initial and subsequent groups consisted of seven novice handball players, while the third group comprised fourteen skilled handball players. All 28 participants were male handball athletes.

To ensure participants’ suitability for the study, all athletes completed a health and injury screening questionnaire before enrollment. None of the participants reported a history of orthopedic, neurological, or neuromuscular disorders, nor any musculoskeletal injuries in the six months preceding the study. In addition, athletes with 2–5 years of handball training experience were excluded to ensure a clear contrast between novice (<2 years) and professional (≥5 years) groups. The novice participants were active in recreational or university-level handball teams, while professional participants were recruited from athletes with continuous competitive training and participation in regional or national leagues. The novice groups (Control group and Test group) consisted of athletes with less than two years of formal training experience, whereas the professional group (Target group) consisted of athletes with at least five years of continuous competitive training. Demographic characteristics, including mean age, height, weight, and body mass index (BMI), are presented in Table 1.

**Figure 1:**
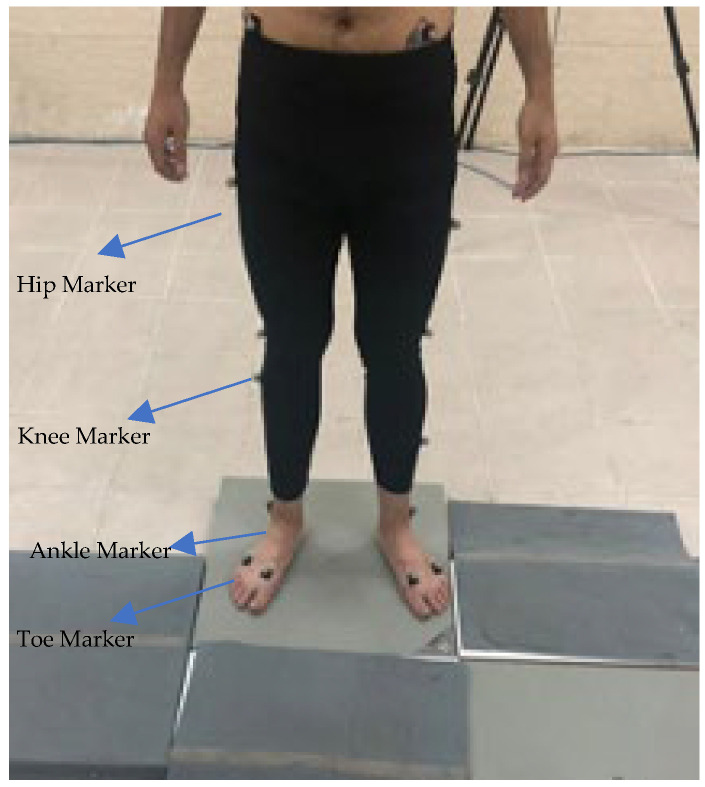
Shows how the markers are placed on different areas of the body.

The initial stage (pre-test) involved administering the backward running test to all individuals from the three groups. In addition, the motion analysis system was used to record the movement of markers placed on the athlete’s body. The system then estimated the movement angles of the ankle, knee, and thigh joints of the supporting leg for athletes in all three groups, namely in the sagittal plane. Following the initial test, both the first and second test groups engaged in a standardized balance training program for a duration of six weeks. Each group participated in three sessions per week, with each session lasting approximately 20 min, resulting in a total of 18 sessions (≈360 min). The training content included single-leg stance exercises, tandem walking, and backward running drills performed on stable surfaces. All sessions were carried out in the motion analysis laboratory under the supervision of a certified coach. Throughout the intervention, participants were instructed not to engage in additional structured handball training or resistance exercise programs outside of the prescribed sessions. Moreover, none of the participants sustained injuries or interruptions during the six-week intervention, and adherence to the training sessions was 100% across groups. The test group performed all drills while blindfolded to reduce their reliance on visual cues, whereas the control group completed the same exercises with normal vision. The third group of professional athletes was studied as the target group. To minimize potential confounding factors, participants were instructed to maintain their regular diet, refrain from additional structured physical training, and avoid stimulant consumption 24 h before each testing session. Moreover, all training sessions were conducted at the same time of day to control for circadian influences. Following the completion of the training course for both groups, the backward running test was repeated, and all data recording and index calculation procedures were repeated. In this study, a total of 18 markers were placed on the lower limbs of all athletes during the initial stage (pre-test) and while running in reverse. Figure 1 depicts the positioning of markers on the various body areas of the athletes.

**Figure 2:**
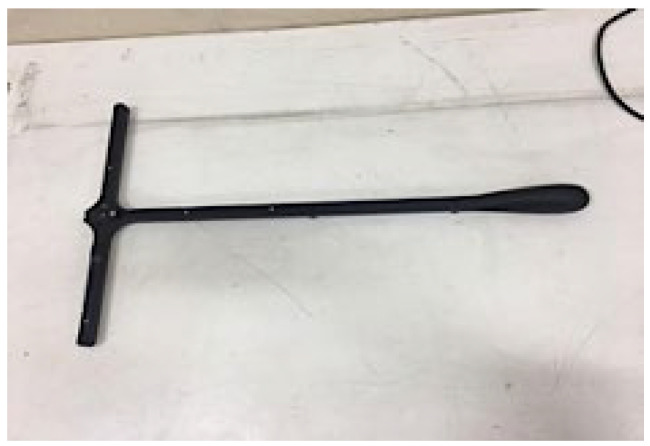
The calibrating equipment utilized throughout the experiment.

The system calibration operation is necessary to enable the usage of cameras, accurately position individuals, and obtain reliable data from the indicators. In order to calibrate, a T-shaped instrument known as a “wand” is employed, which moves continuously within the areas covered by all six cameras until each camera registers a green calibration mark in the software. For the next step, the wand is placed in the center of the screen until all 6 cameras detect the device. Figure 2 depicts the calibrating equipment.

**Figure 3:**
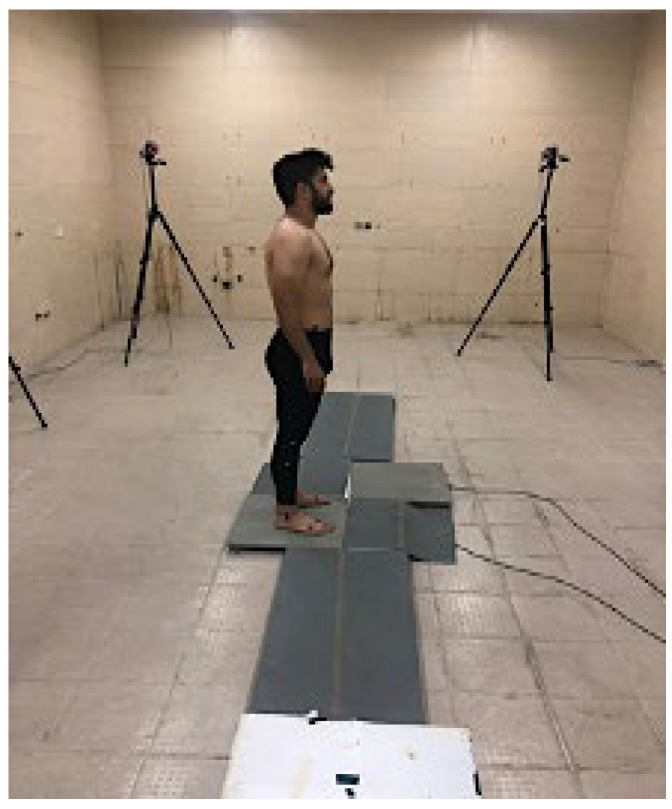
A view of the athlete during the static test.

Following the calibration and marking of the markers on the athlete’s body, a folder named individual was created in the VICON NEXUS software (version 2.9). This software’s measurement section includes the desired items for the lower body, such as height, weight, leg length (from the waist to the heel), and the distance between the lateral bones of the patella and ankle. Following that, the individual is placed on the screen and in front of the camera, and static tests are conducted on him for 15 s. In the calibration phase, the wand is placed on the force plate to precisely calculate the static component without noise. Figure 3 depicts the static test method on one of the athletes.

**Figure 4:**
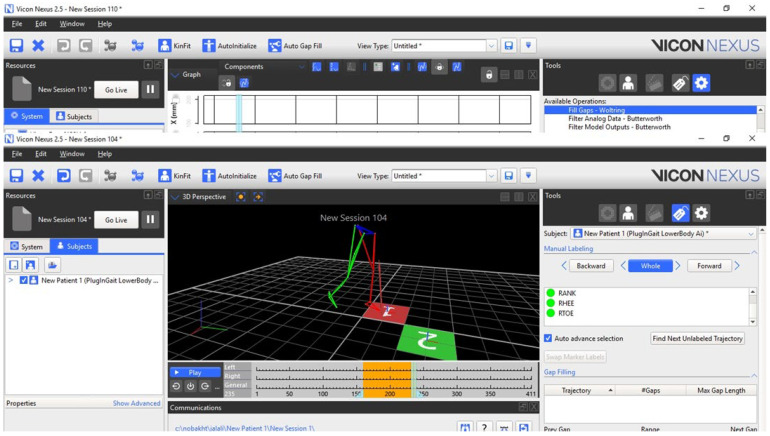
Illustrates the process of navigating in reverse within the software environment.

After completing the static tests, the athlete was instructed to run backwards on the force plate, touching the first and second force plates, respectively. This move was repeated several times for each sample to determine the best option. To obtain good samples, novice athletes typically repeat this movement 15 times. However, for professional athletes, this test produced positive results on average in the first five attempts. Because of the large number of statistical samples, the sampling was initially conducted entirely in the laboratory environment. Following that, the manual labeling approach was used in NEXUS software (ver 20.10) because it provided higher labeling and sampling accuracy than the automatic method. Running the program generates a backward running pattern for the athletes. Figure 4 depicts an athlete’s backward movement pattern in the relevant software environment.

### 2.2. Brief of Mathematical Background

To determine the effectiveness of the exercises presented in both groups and the possibility of recommending the best training method, based on the study conducted by [22,23], the degree of coordination between the organs responsible for backward running was considered:

(1) θRelative Phase=ΦDistal−ΦProximal

In the equation above, θRelative Phase represents the continuous relative phase angle between two organs, while $\Phi_{Distal}$ and $\Phi_{\mathrm{Pr}oximal}$ are the phase angles of the distal and proximal joints, calculated using the phase plane. When the phase angles are calculated, Equation (1) provides the continuous relative phase between two adjacent limbs. In addition, ref. [23] proposed an alternative method for calculating the continuous relative phase between two time series:

(2) $$\begin{array}{l} \theta_{i}=\Phi_{1}(t_{i})-\Phi_{2}(t_{i})\\ \;\;\;\;=\arctan(\frac{{\dot{x}}_{1,norm}(t_{i}).x_{2,norm}(t_{i})-{\dot{x}}_{2,norm}(t_{i}).x_{1,norm}(t_{i})}{x_{1,norm}(t_{i}).x_{2,norm}(t_{i})-{\dot{x}}_{1,norm}(t_{i}).{\dot{x}}_{2,norm}(t_{i})}) \end{array}$$

In this equation, $x_{1}$ and $x_{2}$ are the time series of the common angle, so ${\dot{x}}_{1}$ and ${\dot{x}}_{2}$ are defined as their time derivatives. In this equation, normalized time series are used, which limit the amplitude of the angular movements of the joints’ time series and their first derivative to (1 and −1). This equation equalizes the change range of two-dimensional time series and eliminates the effects of linear trends on signals. Based on this, the angular movements of the joints in various people will be compared, summed, and averaged.

The relative phase indicates the interaction and coordination of distal and proximal organs and joints during activity. It is calculated using four characteristics: joint angular changes and angular velocities. A value of zero means that the joints are completely in phase. Higher values, on the other hand, indicate that non-phase motion is more prevalent. Positive relative phase values indicate that the distal joint is in pre-phase compared to the proximal joint, whereas negative values indicate that the proximal joint is in pre-phase compared to the distal joint. The slope of the relative phase curve also shows which joint moves faster.

A positive slope indicates faster movement of the distal joint in the phase plane, while a negative slope indicates faster movement of the proximal joint. The local maximum and minimum points create a visual representation of the changes in movement coordination dynamics between two joints. The timing of these changes (their position in the desired movement cycle) can distinguish between normal and pathological movements, as well as intergroup movements, and can be used to evaluate the intergroup change process. Continuous relative phase curves should be quantified in order to make statistical comparisons. Thus, the following equations will be used for this purpose:

(3) $$MARP=\frac{\sum_{i=1}^{N}\left|\theta_{i}\right|}{N}$$

The above equation represents the Mean Absolute Relative Phase (MARP), which indicates whether two limbs or joints move in or out of phase. MARP is a relative index in which lower values indicate more in-phase communication between two joints and higher values indicate more out-of-phase communication [24]:

(4) $$DP=\frac{\sum_{i=1}^{N}\left|SD_{i}\right|}{N}\backslash$$

Equation (4) represents the Deviation Phase (DP) [24], which quantifies the extent of variability in the structure of the neuromuscular system and is employed for its evaluation. Moreover, DP serves as a comparative measure: lower values signify a system that is more stable with fewer changes, whereas higher values indicate an unstable neuromuscular system.

Prior to applying parametric tests, data normality was assessed using the Shapiro–Wilk test, and the homogeneity of variances was evaluated using Levene’s test. Both assumptions were satisfied for all variables. The statistical significance threshold was set at α = 0.05. Effect sizes were also calculated to provide a more comprehensive interpretation of the results: Cohen’s d for *t*-tests and eta squared (η^2^) for ANOVA tests. All statistical analyses were performed using SPSS version 21 [25,26].

**Figure 5:**
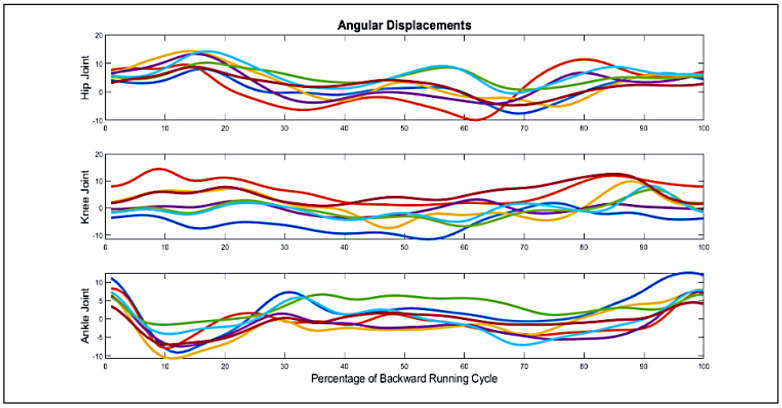
Angular movement curves of the ankle, knee, and pelvic joints of the supporting limb during the test group pretest (balancing exercise with rotating blindfold).

Using the described method, the movement angles of the ankle, knee, and hip joints of the participants’ supporting limbs during backward running were measured with markers, as shown in Figure 5 (The test group’s pretest results). The curves’ range of motion is also calculated in relation to the joint’s neutral positions. According to this diagram, each color corresponds to a single participant.

**Figure 6:**
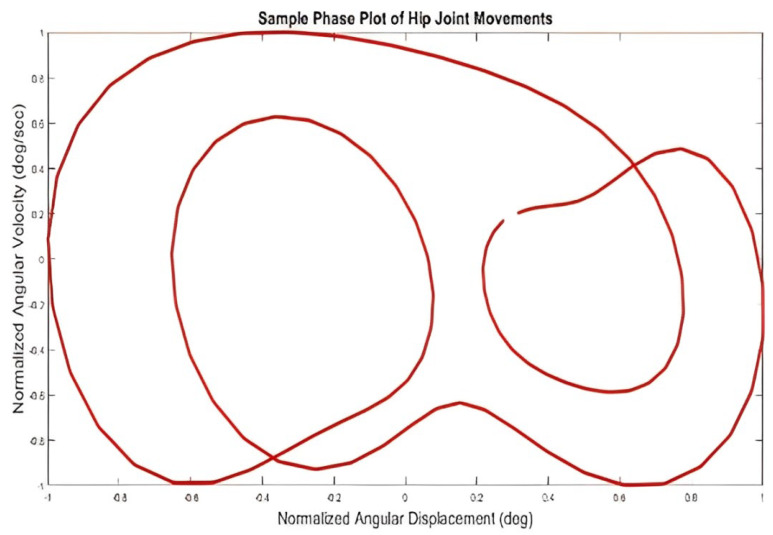
The phase surface curve of a participant’s hip joint.

**Figure 7:**
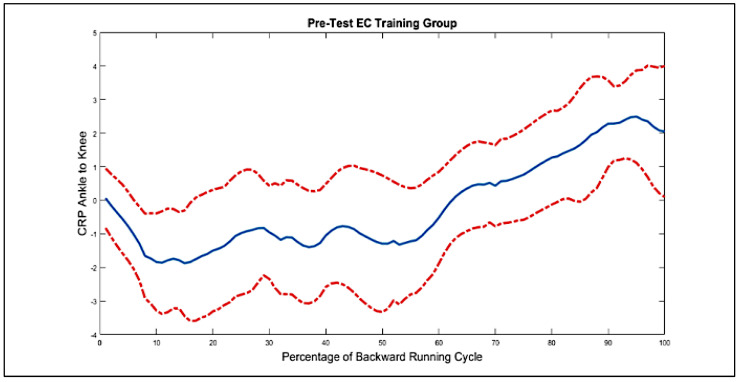
Angular movement curves of the ankle, knee, and pelvic joints of the supporting limb during the test group’s pre-test (balancing exercise with rotating blindfold).

In the following step, the first derivative of the angular position of the joints was calculated, and phase planes were extracted for all of the participants’ joints, one of which is shown in Figure 6. Next, relative phase curves of two adjacent connections were created for each group, one of which is shown in Figure 7. According to this diagram, the blue line represents the average intergroup values of the relative phase at each point in the movement cycle, and the red dashed lines represent the standard deviation.

This study employed the recommended superior training method to assess its efficacy in enhancing body coordination and performance levels, ranging from novice to professional athletes. In order to achieve this objective, descriptive statistics (specifically group mean and variance) were analyzed using classical inferential statistical methods in SPSS version 21 software.

A. An independent *t*-test and one-way analysis of variance were employed to verify that there was no significant disparity between groups in novice athletes and to confirm that there was a significant disparity between novice and professional athletes prior to training.
B. To demonstrate the improvement of inexperienced athletes in both groups following the completion of the prescribed training protocols and to assess the disparity between the groups post-training, paired *t*-test and independent sample *t*-test were employed, respectively.
C. One-way analysis of variance was utilized to assess the disparity between the cohort of inexperienced and seasoned athletes following their training, to gauge the resemblance between novices and professional athletes, and to suggest the optimal training approach.

## 3. Results and Discussion

This section analyzes the research findings both descriptively and analytically, with the descriptive analysis presented in the form of values of central tendency, dispersion, and shapes.

### 3.1. Results of Descriptive Statistics Calculations

Using the equations, average MARP and average DP were calculated for each group and stage. Table 2 and Table 3 show the results for average MARP and average DP, respectively.

**Figure 8:**
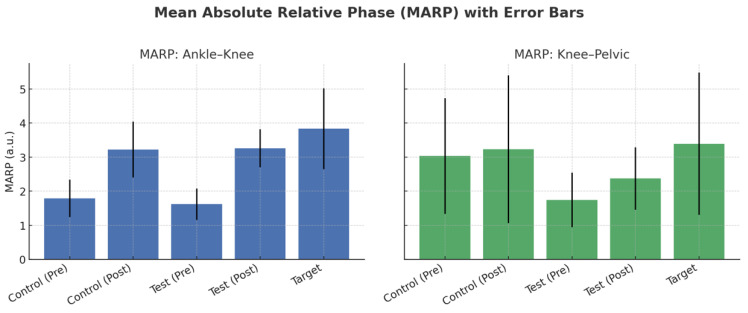
MARP values for ankle–knee (**left**) and knee–pelvic (**right**) couplings across groups. Error bars indicate standard deviations. Control and test groups are shown for both pre-test and post-test stages, while the target group (professional players) is shown as a single reference test.

**Figure 9:**
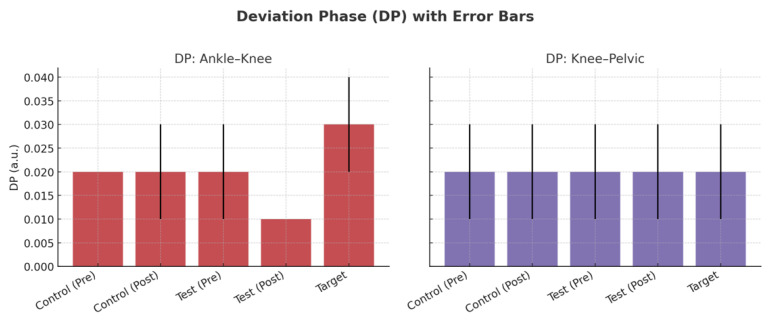
DP values for ankle–knee (**left**) and knee–pelvic (**right**) couplings across groups. Error bars indicate standard deviations. Control and test groups are shown for both pre-test and post-test stages, while the target group (professional players) is shown as a single reference test.

Figure 8 displays the MARP values for ankle–knee and knee–pelvic couplings across groups. At baseline, professionals exhibited descriptively higher ankle–knee MARP compared with novices. After training, both novice groups showed increased ankle–knee MARP values, approaching the professional profile, whereas knee–pelvic MARP values exhibited smaller changes. As illustrated in Figure 8, error bars highlight the variability in each group, and the figure provides a clearer depiction of between-group differences compared with the tabulated values. Figure 9 presents the DP values for ankle–knee and knee–pelvic couplings. As shown in Figure 9, DP values remained largely stable across groups and stages. While professional players displayed slightly higher ankle–knee DP compared with novices, these differences were not statistically significant. Knee–pelvic DP values were similar across all groups, indicating that coordinative variability was less sensitive to the short-term training intervention. Accordingly, Figure 3 complements the MARP findings presented in Figure 8 by showing that variability measures were less responsive to training than coordination measures.

According to Table 2 and Table 3, the MARP index between the ankle and knee joints before training for the control and test groups is 1.79 and 1.62, respectively, whereas it is 3.83 for professional athletes. Overall, it is possible to conclude that professional athletes’ ankle and knee joints function more out of phase than those of novice athletes. In other words, professional athletes demonstrate this ability when backward running. In addition, the MARP index showed a statistically significant difference between the test and control groups. For clarity, the three study groups are defined as follows:

- Test Group: novice players who performed balance training with a blindfold.
- Control Group: novice players who performed the same training without a blindfold (normal vision).
- Target Group: professional players with regular training experience.

### 3.2. Comparison of Motor Coordination Results of Two Control and Test Groups in the Pre-Test

For this comparison, the subjects’ motor coordination was assessed using the MARP and DP indices and an independent samples *t*-test to ensure that there was no significant difference between the two groups prior to training. To ensure there was no significant difference between the two groups before training, the independent *t*-test was conducted, and the results (*t*-values, degrees of freedom, and *p*-values) are presented in Table 4. According to this table, Levin’s hypothesis of equal variances in all indicators was rejected, and the variance of all indicators was found to be equal between two groups.

According to Table 4, there is no noticeable disparity between the two groups prior to the exercises, and both groups exhibit comparable levels of organ coordination. Specifically, ankle–knee MARP did not differ between groups (t(12) = 0.64, *p* = 0.53), knee–pelvic MARP (t(12) = 1.75, *p* = 0.10), ankle–knee DP (t(12) = 0.37, *p* = 0.72), and knee–pelvic DP (t(12) = 1.21, *p* = 0.24) were also non-significant. The subsequent analysis presents the assessment of the disparity in motor coordination outcomes between the control and test groups compared to the target group prior to commencing the exercises. In order to assess the disparity in limb movement coordination between novice athletes (control and test group) and professional athletes (target group), A one-way ANOVA test was conducted using MARP and DP indices. The results, including F-values, degrees of freedom, and *p*-values, are shown in Table 5. Ankle–knee MARP showed a significant group effect (F(2,25) = 9.42, *p* < 0.001), as did ankle–knee DP (F(2,25) = 7.85, *p* < 0.01), whereas knee–pelvic MARP (F(2,25) = 1.98, *p* = 0.15) and knee–pelvic DP (F(2,25) = 1.87, *p* = 0.16) did not.

According to Table 5, ankle–knee MARP showed a significant group effect (F(2,25) = 9.42, *p* < 0.001), as did ankle–knee DP (F(2,25) = 7.85, *p* < 0.01), whereas knee–pelvic MARP (F(2,25) = 1.98, *p* = 0.15) and knee–pelvic DP (F(2,25) = 1.87, *p* = 0.16) did not. To determine the difference between the groups, the post hoc test described in Table 6 was used.

Post hoc tests in Table 6, confirmed that professionals differed from both novice groups in ankle–knee MARP (test vs. target: t(19) = 4.76, *p* < 0.001; control vs. target: t(19) = 4.85, *p* < 0.001) and in ankle–knee DP (test vs. target: t(19) = 2.48, *p* = 0.02; control vs. target: t(19) = 2.78, *p* = 0.01). Differences between the two novice groups were non-significant across indices (all *p* > 0.38). Knee–pelvic coordination indices showed no significant pairwise differences (all *p* ≥ 0.13).

According to the results presented in Table 3 and Table 6, the following points can be made: The DP index, which is used as a stability index, is 0.02 in the control and test groups, and 0.03 in the target group while performing the exercise of backward running in ankle and knee joints prior to training. The follow-up test revealed the difference between the control group and the test group with the target group. This result can be explained in two ways: First, professional athletes exhibit greater instability than novice athletes when performing the task of backward running. This explanation contradicts the purpose of this study. We assumed that professional athletes perform better when running backward, so this study was designed to improve the performance of novice athletes. According to dynamic systems theory, professional athletes have a more flexible movement pattern and greater joint maneuverability by increasing their degree of freedom when backward running (less in-phase performance based on the MARP index), and thus their DP index is higher. On the other hand, it could be argued that novice athletes have more rigid movement patterns and tend to consciously control joint movements and coordination of ankle and knee joints, reducing the degree of freedom of the neurological system. This assumption does not contradict the study’s initial hypothesis of greater diversity in skilled systems, because the variability of dynamic systems is evaluated from both a quantitative and a structural perspective, and this structural variability must be evaluated by repeating the movement cycle a sufficient number of times, which is not possible in the laboratory setting. It is suggested that this issue be investigated in future research using wearable sensors while backward running at various speeds for different groups of athletes.

To compare the results of movement coordination in each group after the end of the exercises, and to evaluate the exercise’s effectiveness, the indicators before and after the exercise were compared using the paired *t*-test. The results of this test are shown in Table 7.

Table 7 compares two groups of novice athletes before and after training. According to Table 7, In the control group, ankle–knee MARP increased significantly (t(6) = 5.02, *p* < 0.001), whereas knee–pelvic MARP (t(6) = 0.19, *p* = 0.88) and both DP indices (ankle–knee: t(6) = 1.56, *p* = 0.16; knee–pelvic: t(6) = 0.85, *p* = 0.43) did not change significantly. In the test group, ankle–knee MARP also increased (t(6) = 4.72, *p* < 0.001), while knee–pelvic MARP (t(6) = 1.38, *p* = 0.21) and DP indices (ankle–knee: t(6) = 0.82, *p* = 0.44; knee–pelvic: t(6) = 1.09, *p* = 0.31) remained non-significant. We can conclude that non-phase performance in this motor coordination has improved, and that the performance of novice athletes has now approached that of professional athletes in the target group. However, the findings revealed that stability (or flexibility) during the task did not change statistically following training. The results show that the MARP index increased significantly after training, indicating improved motor coordination between the ankle and knee joints. Similarly, MARP for the knee and hip joints improved following exercise, but the difference was not statistically significant. Furthermore, as shown in the table, no joint group’s DP index changed before or after training. In the control group, ankle–knee MARP increased significantly (t(6) = 5.02, *p* < 0.001), whereas knee–pelvic MARP (t(6) = 0.19, *p* = 0.88) and both DP indices (ankle–knee: t(6) = 1.56, *p* = 0.16; knee–pelvic: t(6) = 0.85, *p* = 0.43) did not change significantly. In the test group, ankle–knee MARP also increased (t(6) = 4.72, *p* < 0.001), while knee–pelvic MARP (t(6) = 1.38, *p* = 0.21) and DP indices (ankle–knee: t(6) = 0.82, *p* = 0.44; knee–pelvic: t(6) = 1.09, *p* = 0.31) remained non-significant.

A one-way ANOVA test was used to assess the effectiveness of each group as well as the interaction between the joints of novice and professional athletes, and the results are fully reported in Table 8.

After training, group differences were non-significant for ankle–knee MARP (F(2,25) = 1.24, *p* = 0.30) and knee–pelvic MARP (F(2,25) = 0.68, *p* = 0.51). Ankle–knee DP showed a group effect (F(2,25) = 5.12, *p* = 0.01), whereas knee–pelvic DP did not (F(2,25) = 1.74, *p* = 0.19). To detect the difference between groups, a post hoc test was used, the results of which are shown in Table 9. For ankle–knee DP, the test group differed from professionals (t(19) = 3.12, *p* = 0.01), while control vs. test (t(12) = 1.80, *p* = 0.10) and control vs. professionals (t(19) = 0.45, *p* = 0.65) were non-significant.

Table 8 and Table 9 show that novice athletes in both the control and test groups have movement coordination comparable to that of professional athletes. Table 6 and Table 7 show a statistically significant difference in MARP index in ankle and knee coordination before exercise; however, there is no significant statistical difference after training. The results also show a statistically significant difference in ankle and knee DP index between the test group (novice athletes who receive additional blindfold exercises) and the target group (professional athletes). Finally, using a one-way ANOVA test, the controllability and coordination of ankle movements were compared between professional and novice athletes in the presence of environmental disturbances. This evaluation is shown in Table 10.

In disturbed environments (Table 10), descriptive differences favored professionals over novices—e.g., ankle–knee MARP (0.63 vs. 0.42) and ankle–knee DP (0.59 vs. 0.14), as well as knee–pelvic MARP (0.48 vs. 0.31) and DP (0.53 vs. 0.23). However, none of these differences reached statistical significance (ankle–knee MARP: F(1,19) = 0.58, ***p*** = 0.63; knee–pelvic MARP: F(1,19) = 0.50, ***p*** = 0.48; ankle–knee DP: F(1,19) = 0.30, ***p*** = 0.59; knee–pelvic DP: F(1,19) = 1.25, ***p*** = 0.53). These results suggest higher ankle-movement controllability descriptively in professionals, but without inferential support at α = 0.05.

### 3.3. Interpretation and Practical Applications

Locomotion is the primary component of the process of human evolution. Motor skills are not innate in humans, but rather acquired through learning. While it is indeed true that certain human movements can be instinctual or formed by natural reactions, it is important to note that these movements can also be acquired and mastered through learning. The primary objective of human learning in relation to movement skills is to execute them with accuracy, precision, and efficiency. Furthermore, individuals seek details regarding the nature of the movement and its dynamics, including its evolution and transformations. One effective approach to acquiring knowledge and information about movement acquisition is to combine knowledge of movement control with the field of biomechanics. In the sport of handball, transitioning from an offensive to a defensive position requires the athletes to move in a backward direction [27]. Given the visual system’s inability to be utilized, athletes rely on their proprioceptive sense in this scenario. In contrast, backward running exhibits distinct biomechanical traits compared to forward running. The knee and hip joint coordination before training among three groups is compared, and the results are presented in Table 2 and Table 3. Table 5 and Table 6 do not demonstrate a statistically significant distinction in terms of MARP and DP indices among the three groups (target, control, and test) even prior to training. This lack of significance may be attributed to the specific muscles engaged in the joints, as well as the involvement of the ankle and leg in running and motor neuron settings, as suggested by [28,29]. Nevertheless, the precise mechanisms by which individuals consciously manage and regulate joint stiffness remain uncertain [30,31]. Sports such as handball and trail running are at high risk of lower extremity injuries, especially to the knee, ankle, and Achilles’ tendon. Several factors contribute to the occurrence of these injuries. The findings of this study could help design effective prevention and treatment programs for athletes [32]. In addition, complete statistical information (test statistics, degrees of freedom, and *p*-values) has been reported in all tables to ensure clarity and transparency of the findings.

An important implication of these findings is their relevance to injury prevention. Handball involves frequent backward running and sudden changes in direction, which increase the risk of injuries to the ankle, knee, and Achilles’ tendon. The improvements in inter-joint coordination observed in both novice groups suggest that proprioceptive-based training, including blindfolded balance and backward running drills, may enhance joint stability and reduce susceptibility to lower-limb injuries. These results support the integration of proprioceptive training elements into handball conditioning programs to minimize injury risk and promote safer performance [32,33,34].

This research examines the use of wearable sensors and bionic motion sensors to assess backward running. This research used bionics to mimic a computer for a more comprehensive understanding of bodily motions, using digital body models to assess forces during backward running. The collected data and approach may provide insights that could be explored in future research, such as in the design of virtual reality training programs or the development of intelligent insoles and motion sensors. However, these potential applications go beyond the scope of the present short-term study [29,30]. Our use of independent *t*-tests, paired *t*-tests, and ANOVA is consistent with previous coordination studies, for example, Galgon & Shewokis, who analyzed MARP and DP indices using these tests, and Ippersiel et al., who applied similar statistical procedures in spinal coordination analysis [24,25].

### 3.4. Limitations and Future Directions

This study has some limitations that should be considered when interpreting the results. First, the sample size was relatively small (28 participants), which may limit the generalizability of the findings to larger athlete populations. Second, the intervention period was limited to six weeks, and therefore, the present results only reflect short-term effects. The potential long-term impacts of blindfold training on coordination and flexibility could not be evaluated in this study. Third, although participants were instructed to maintain their regular diet, refrain from additional training, and avoid stimulant consumption, uncontrolled lifestyle factors such as sleep quality, nutrition, and previous training history may have acted as confounding variables influencing the outcomes. Future research should therefore recruit larger and more diverse samples, extend the training period to evaluate long-term effects, and control or measure lifestyle-related confounders more systematically.

Recent advances in artificial intelligence and machine learning open promising avenues for analyzing complex movement patterns in sports science. AI-assisted approaches have been successfully applied in diverse domains such as media literacy research [35], distributed multi-agent learning [36], aircraft design optimization [37], sustainable architecture [38], smart built environments [39], and ethical frameworks for technology integration [40]. Other contributions highlight the role of AI in blockchain-based food supply chains [41], digitalization and mental health [42], diagnosis and patient care [43], healthcare supply chains [44], health tourism [45], energy systems [46], and even clinical dentistry [47]. These examples demonstrate the versatility of AI and suggest that future investigations of backward and forward movements in handball could benefit from machine learning techniques to enhance training design, injury prevention, and rehabilitation applications.

Among the applications of this study, the benefits for handball players can be summarized as follows: increasing agility and coordination of movement, promoting effective defense, preventing injuries, designing corrective exercises, more accurate training planning and increasing functional endurance. This study can also be very useful in the research and development of sports technologies. The backward motion data in this study can also be used in the development of wearable systems, motion analysis applications and training tools for coaches and athletes.

## 4. Conclusions

This study showed that professional handball players exhibit more adaptive inter-joint coordination patterns compared to novices. Short-term proprioceptive training, both with and without blindfolds, improved ankle–knee coordination in novices, moving them closer to the professional coordination profile. These findings suggest that proprioceptive-based drills can be integrated into handball training routines to enhance motor control, improve performance, and reduce the risk of lower-limb injuries. Coaches and practitioners can apply such evidence-based strategies to design training programs that improve coordination and resilience in athletes.

Therefore, the present short-term results do not provide sufficient evidence to confirm or reject the long-term benefits of additional blindfold training on motor flexibility. Further longitudinal studies are required to clarify this. The results may have practical implications for the design of short-term training and rehabilitation protocols for handball players and other athletes involved in similar dynamic movement patterns. However, long-term effects remain to be investigated.

## Figures and Tables

**Table 1:** Demographic characteristics of study participants.

Group	n	Age (Years)	Height (m)	Weight (kg)	BMI	Gender	Training Experience
Group #1 (Novice)	**7**	**21**	**1.70**	**75**	**25.95**	**Male**	**<2 years**
Group #2 (Novice)	**7**	**22**	**1.75**	**73**	**23.83**	**Male**	**<2 years**
Group #3 (professional)	**14**	**21**	**1.72**	**74**	**24.01**	**Male**	**≥5 years**
**Mean**	**-**	**21.3**	**1.72**	**74**	**24.60**	**Male**	**-**
**Std. Deviation**	**-**	**-**	**0.02**	**0.81**	**0.95**	**-**	**-**

**Table 2:** Shows the absolute mean values of continuous relative phase for the target, control, and test.

Group	Stage	Relative Phase Between Limbs	MARP Mean
**control group**	**pre-test**	**ankle to knee**	79 (0.55)
		**knee to pelvic**	3.03 (1.70)
	**final test**	**ankle to knee**	3.22 (0.82)
		**knee to pelvic**	3.23 (2.17)
**test group**	**pre-test**	**ankle to knee**	1.62 (0.46)
		**knee to pelvic**	1.74 (0.80)
	**final test**	**ankle to knee**	3.26 (0.56)
		**knee to pelvic**	2.37 (0.92)
**target group**	**single test**	**ankle to knee**	3.83 (1.19)
		**knee to pelvic**	3.39 (2.09)

**Table 3:** Shows the standard deviation values of continuous relative phase for the target, control, and test.

Group	Stage	Relative Phase Between Limbs	DP Mean
control group	pre-test	ankle to knee	**0.02 (0.00)**
		knee to pelvic	**0.02 (0.01)**
	final test	ankle to knee	**0.02 (0.01)**
		knee to pelvic	**0.02 (0.01)**
test group	pre-test	ankle to knee	**0.02 (0.01)**
		knee to pelvic	**0.02 (0.01)**
	final test	ankle to knee	**0.01 (0.00)**
		knee to pelvic	**0.02 (0.01)**
target group	single test	ankle to knee	**0.03 (0.01)**
		knee to pelvic	**0.02 (0.01)**

**Table 4:** Presents the independent samples *t*-test results, including t-values, degrees of freedom, and *p*-values for the continuous relative phase between the ankle–knee and knee–pelvic.

Index	Relative Phase Between Limbs	*p*-Value	t(df)
MARP	ankle to knee	0.53	t(12) = 0.64
	knee to pelvic	0.10	t(12) = 1.75
DP	ankle to knee	0.72	t(12) = 0.37
	knee to pelvic	0.24	t(12) = 1.21

**Table 5:** Presents the one-way ANOVA results (F-values, degrees of freedom, and *p*-values) of the continuous relative phase for the target, control, and test groups before training.

Index	Relative Phase Between Limbs	*p*-Value	F(df1,df2)
**MARP**	ankle to knee	0.00	F(2,25) = 9.42
	knee to pelvic	0.15	F(2,25) = 1.98
**DP**	ankle to knee	0.00	F(2,25) = 7.85
	knee to pelvic	0.16	F(2,25) = 1.87

**Table 6:** Shows the results of an ANOVA post hoc statistical test between the target, control, and test groups before training.

Index	Relative Phase Between Limbs	Comparison Group No.1	Comparison Group No.2	*p*-Value	t(df)
**MARP**	**ankle to knee**	**test group**	**control group**	0.93	t(12) = 0.08
			**target group**	0.00	t(19) = 4.76
		**control group**	**test group**	0.93	t(12) = 0.93
			**target group**	0.00	t(19) = 4.85
		**target group**	**test group**	0.00	t(19) = 4.76
			**control group**	0.00	t(19) = 4.85
	**knee to pelvic**	**test group**	**control group**	0.38	t(12) = 0.91
			**target group**	0.13	t(19) = 1.63
		**control group**	**test group**	0.38	t(12) = 0.91
			**target group**	0.90	t(19) = 0.90
		**target group**	**test group**	0.13	t(19) = 1.63
			**control group**	0.90	t(19) = 0.13
**DP**	**ankle to knee**	**test group**	**control group**	0.96	t(12) = 0.05
			**target group**	0.02	t(19) = 2.48
		**control group**	**test group**	0.96	t(12) = 0.05
			**target group**	0.01	t(19) = 2.78
		**target group**	**test group**	0.02	t(19) = 2.48
			**control group**	0.01	t(19) = 2.78
	**knee to pelvic**	**test group**	**control group**	0.41	t(12) = 0.84
			**target group**	0.14	t(19) = 1.52
		**control group**	**test group**	0.41	t(12) = 0.84
			**target group**	0.87	t(19) = 0.12
		**target group**	**test group**	0.14	t(19) = 1.52
			**control group**	0.87	t(19) = 0.12

**Table 7:** Summarizes the paired *t*-test results, including *t*-values, degrees of freedom, and *p*-values, comparing the control and test groups before and after training.

Group	Index	Relative Phase Between Limbs	*p*-Value	t(df)
**control group**	**MARP**	**ankle to knee**	0.00	t(6) = 5.02
		**knee to pelvic**	0.88	t(6) = 0.19
	**DP**	**ankle to knee**	0.16	t(6) = 1.56
		**knee to pelvic**	0.43	t(6) = 0.85
**test group**	**MARP**	**ankle to knee**	0.00	t(6) = 4.72
		**knee to pelvic**	0.21	t(6) = 1.38
	**DP**	**ankle to knee**	0.44	t(6) = 0.82
		**knee to pelvic**	0.31	t(6) = 1.09

**Table 8:** Presents the one-way ANOVA results (F-values, degrees of freedom, and *p*-values) comparing the control, test, and target groups after training.

Index	Relative Phase Between Limbs	*p*-Value	F(df1,df2)
MARP	ankle to knee	0.30	**F(2,25) = 1.24**
	knee to pelvic	0.51	**F(2,25) = 0.68**
DP	ankle to knee	0.01	**F(2,25) = 5.12**
	knee to pelvic	0.19	**F(2,25) = 1.74**

**Table 9:** Provides the post hoc comparisons of the one-way ANOVA results, including t-values, degrees of freedom, and *p*-values, among the control, test, and target groups after training.

Index	Relative Phase Between Limbs	Comparison Group No.1	Comparison Group No.2	*p*-Value	t(df)
MARP	ankle to knee	test group	control group	1.00	t(12) = 0.00
			target group	0.43	t(12) = 0.80
		control group	test group	1.00	t(12) = 0.00
			target group	0.38	t(19) = 0.80
		target group	test group	0.43	t(19) = 0.80
			control group	0.38	t(19) = 0.80
	knee to pelvic	test group	control group	0.68	t(12) = 0.42
			target group	0.49	t(19) = 0.70
		control group	test group	0.68	t(12) = 0.42
			target group	0.98	t(19) = 0.02
		target group	test group	0.149	t(19) = 1.50
			control group	0.98	t(19) = 0.02
DP	ankle to knee	test group	control group	0.10	t(12) = 1.80
			target group	0.01	t(19) = 3.12
		control group	test group	0.10	t(12) = 1.80
			target group	0.65	t(19) = 0.45
		target group	test group	0.01	t(19) = 3.12
			control group	0.65	t(19) = 0.45
	knee to pelvic	test group	control group	0.78	t(12) = 0.28
			target group	0.56	t(19) = 0.60
		control group	test group	0.78	t(12) = 0.28
			target group	0.18	t(19) = 0.18
		target group	test group	0.56	t(19) = 0.60
			control group	0.18	t(19) = 1.42

**Table 10:** Presents the one-way ANOVA results, including F-values, degrees of freedom, and *p*-values, for the controllability and coordination of ankle movements between professional (target) and novice (test) athletes in disturbed environments.

Index	Controllability and Coordination of Ankle Movements	*p*-Value	
		Test Group	Target Group
		F(df1,df2)	
MARP	ankle to knee	**0.42**	**0.63**
		**F(1,19) = 0.58**	
	knee to pelvic	**0.31**	**0.48**
		**F(1,19) = 0.50**	
DP	ankle to knee	**0.14**	**0.59**
		**F(1,19) = 0.30**	
	knee to pelvic	**0.23**	**0.53**
		**F(1,19) = 1.25**	

## Data Availability

No new data were created or analyzed in this study.
